# The rationale and plan for creating a World Antimalarial Resistance Network (WARN)

**DOI:** 10.1186/1475-2875-6-118

**Published:** 2007-09-06

**Authors:** Carol Hopkins Sibley, Karen I Barnes, Christopher V Plowe

**Affiliations:** 1 Department of Genome Sciences, University of Washington, Seattle, WA, 98195-5065 USA; 2 Division of Clinical Pharmacology, Department of Medicine, University of Cape Town, Cape Town, South Africa; 3 Center for Vaccine Development, University of Maryland School of Medicine, 685 West Baltimore Street, HSF1-480, Baltimore, Maryland 21201, USA

## Abstract

Drug resistant malaria was a major factor contributing to the failure of a worldwide campaign to eradicate malaria in the last century, and now threatens the large investment being made by the global community in the rollout of effective new drug combinations to replace failed drugs. Four related papers in this issue of Malaria Journal make the case for creating the World Antimalarial Resistance Network (WARN), which will consist of four linked open-access global databases containing clinical, in vitro, molecular and pharmacological data, and networks of reference laboratories that will support these databases and related surveillance activities. WARN will serve as a public resource to guide antimalarial drug treatment and prevention policies and to help confirm and characterize the new emergence of new resistance to antimalarial drugs and to contain its spread.

## Background

In the mid-20^th ^century, highly effective treatment and prophylaxis for *Plasmodium falciparum *malaria with a single safe and inexpensive drug, chloroquine, was available worldwide. For more than a decade, no resistance to chloroquine was seen, leading to optimism that none would arise, and chloroquine and DDT formed the pillars of a worldwide campaign to eradicate malaria [1]. After it did emerge in Southeast Asia and South America, resistance to chloroquine spread globally, contributing to the abandonment of the eradication effort and leading to large increases in malaria morbidity and mortality [2]. Resistance to the next generations of drugs, first sulphadoxine-pyrimethamine (SP) and then mefloquine, quickly followed their introduction and severely compromised their efficacy [3-5]. Long after drug resistant malaria has become a public health crisis in much of the developing world, passionate advocacy for replacing ineffective monotherapies with new combination therapies designed to deter resistance is finally being heard and acted upon [6,7]. Artemisinin-based combination therapies (ACTs) combine short-acting, highly efficacious artemisinin derivatives with longer acting partner drugs in an approach similar to that used to combat drug resistant tuberculosis and HIV.

Decisions to change malaria treatment policies are usually made at a national level without consideration of local differences in drug efficacy and without serious efforts to coordinate strategies for deterring resistance on a regional or global level [8]. With a few notable exceptions, the results of this uncoordinated approach have been suboptimal at best, and arguably catastrophic. Decisions have been made based on insufficient evidence, and treatment policy changes have been made too late and implemented too ineffectively, at a huge cost to health and life in addition to the heavy financial burden to households and healthcare providers.

The high levels of support and enthusiasm accompanying the roll-out of ACTs create an historic opportunity to get it right this time. The transition from 20^th ^century monotherapies to 21^st ^century ACTs has spurred a massive increase in the number of studies of clinical drug efficacy being conducted, many accompanied by the research on genetic correlates of drug resistant malaria and the changes of *in vitro *responses of *P. falciparum *to antimalarial drugs. Moreover, the relationship between the pharmacokinetics and treatment response is being elucidated for key antimalarials The World Health Organization has recently published a compendium of these data [9] and a number of meta-analyses have been published [10-13]. However, the many differences among the studies in design, execution and analysis, and especially in formats for recording and reporting data, reduce greatly the utility of these pooled datasets [14,15]. In addition, access is frequently limited to the summary data, and data from unpublished studies is often not available. Thus, a new approach to collation, analysis and presentation of the data on drug resistant malaria is urgently needed.

More than 50 countries have changed their national policy to recommend ACTs as the first line treatment for falciparum malaria, but in many malaria endemic countries both financial and practical difficulties have slowed the implementation of these new policies. There is vigorous debate about which drug combinations are the most suitable and how these new treatments should be deployed and funded. In this context, it is of paramount importance to policymakers, funding bodies and researchers, to document the clinical efficacy of existing treatments and establish an ongoing surveillance system to monitor the continued efficacy of new antimalarials. Moreover, there is little doubt that resistance will eventually evolve, even to the ACTs. When this happens, the earliest possible warning of resistance to either the artemisinin derivatives or their partner drugs will be the key to avoiding the disaster that would entail the loss of this pivotal class of drugs. It is even possible that a sensitive global surveillance system for ACT resistance could guide strategies to contain and deter resistance.

## The plan

Four papers in this issue propose the creation of a web-based World Antimalarial Resistance network (WARN) that would be accessible to all users. This comprehensive efficacy and resistance database will provide malaria control managers, surveillance programs and policymakers with prompt access to up-to-date evidence of temporal and geographic trends in antimalarial drug resistance at the global scale. By detecting early indications of resistance, the database will permit focused epidemiological and parasitological investigations to confirm, or importantly, refute, suspected antimalarials drug resistance, providing evidence to support the case for or against a change in treatment policy. In addition, it would inform optimal utilization of antimalarials and facilitate the longest possible useful therapeutic life of the available antimalarial drugs and drug combinations.

These goals require a robust, prompt, accessible, flexible and comprehensive collation of high quality data on antimalarial drugs therapeutic efficacy, molecular and in vitro markers of resistance, and the relationship between drug dose, concentration and treatment response.

The proposed database will be modular and incorporate data of four different kinds: clinical drug efficacy, definition and prevalence of molecular markers of resistance to particular drugs, in vitro response of reference and clinical isolates to drugs under study, and the pharmacokinetic and pharmacodynamic properties of these drugs in important target populations. It will include tools that facilitate keying and entering the data into the database, and tools that will enable sophisticated analysis and output of the data in formats useful to a wide variety of users. These tools will be freely available to all in the community, and should facilitate the preparation of data for publication. The goal is to provide a timely and efficient locus for all groups involved in the determination of drug responses to *P. falciparum *to access and share data that are relevant to this important issue. The creation and maintenance of the database will provide significant opportunities for development of local capacity for use of tools of analysis and presentation of data collected in a wide variety of settings.

**Figure 1:**
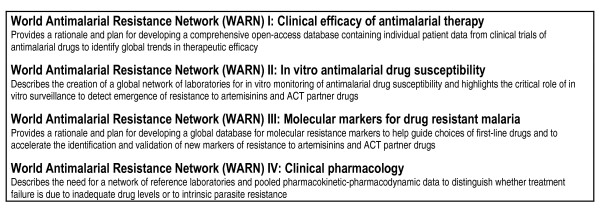
List of papers in this issue describing creation of global clinical, in vitro, molecular and clinical pharmacology databases comprising a World Antimalarial Resistance Network (WARN).

The four papers that follow (Figure 1) make the case for a WARN organized by the four types of data it would incorporate: clinical, molecular, in vitro and pharmacokinetic; and propose a framework for establishment and use of information in each module and for the linkage of the information between modules.

## Authors' contributions

CHS wrote the manuscript and KIB and CVP contributed significant editorial input. All authors have read and approved the manuscript.
